# Non-surgical periodontal therapy with and without hyaluronic acid gel in type 2 diabetic stage-II periodontitis patients: a randomized clinical trial

**DOI:** 10.1186/s12903-025-06485-2

**Published:** 2025-07-15

**Authors:** Reem Al-Abbadi, Nesma Shemais, Alaa Nawwar, Karim M. Fawzy El-Sayed

**Affiliations:** 1 Oral Medicine & Periodontology Department, Faculty of Dentistry, Cairo University, Al Saraya Str. 11, Manial, Cairo, Egypt; 2 Oral and Maxillofacial Radiology Department, Faculty of Dentistry, Cairo University, Cairo, Egypt; 3 Oral and Maxillofacial Radiology Department, School of Dentistry, New Giza University, Giza, Egypt; 4 Clinic for Conservative Dentistry and Periodontology, School of Dental Medicine, Christian Albrechts University, Kiel, Germany; 5 Stem Cells and Tissue Engineering Research Unit, Faculty of Dentistry, Cairo University, Cairo, Egypt

**Keywords:** Periodontics, Hyaluronic acid, Professional mechanical plaque removal, Periodontitis, Diabetes

## Abstract

**Background:**

The present trial evaluated clinically and radiographically the effect of topically applied hyaluronic acid (HA) gel in conjunction with professional mechanical plaque removal (PMPR) in type 2 diabetic stage-II periodontitis patients.

**Methodology:**

26 controlled (HbA1c < 7%) type 2 diabetic stage-II grade B periodontitis patients were included in the current trial and randomly assigned to test (*n* = 13 patients; PMPR + HA) or control (*n* = 13; PMPR) groups. At baseline, three- and six-months clinical attachment level (CAL; primary outcome), probing pocket depth (PPD), gingival recession depth (GRD), bleeding on probing (BOP), O’Leary plaque index (PI), HbA1c level, radiographic bone density (RBD) and defect depth (DD; all secondary outcomes) were assessed.

**Results:**

Although CAL, PPD, BOP, PI, DD and RBD independently significantly improved in the PMPR + HA and the PMPR groups (*p* < 0.05), no significant differences were notable between both groups. HbA1c significantly decreased solely in the PMPR + HA group (*p* < 0.05).

**Conclusion:**

Type 2 diabetic patients with stage-II periodontitis, benefit clinically from PMPR in the presence or absence of adjunctive HA gel application. Interestingly, HA as an adjunct to PMPR significantly improved HbA1c levels in controlled type 2 diabetic stage-II periodontitis patients.

**Trial registration:**

The study was registered in the US National Institutes of Health Clinical Trials Registry (NCT05543434) in September 2022.

## Background

Periodontitis is a multi-factorial inflammatory disorder of the teeth supporting structures associated with microbial dysbiosis [1, 2], hallmarked, untreated, by a progressive loss of the alveolar bone, the formation of periodontal pockets and gingival bleeding [3]. Diabetes mellitus (DM) is bi-directionally associated with periodontitis representing one of its major risk factors. Periodontitis is documented to negatively influence the glycemic control [4, 5] and increase the severity of diabetic complications [6], while uncontrolled DM could foster a more aggressive progression of periodontal disease [7].

Professional mechanical plaque removal (PMPR), with its anti-infective supra- and subgingival mechanical tooth surface instrumentation [8], is considered essential during the second phase of stages I–IV periodontitis for effective periodontal therapy [9, 10]. Yet, although successful PMPR can lower inflammation surrogate parameters [11, 12], certain periodontally affected sites and/or patients may not respond well, with a failure to transform the dysbiotic periodontopathic subgingival biofilm into a symbiotic one [13] and a persistence of a chronic inflammatory reaction.

Being a pivotal component of all naturally occurring extracellular matrices in the human body, hyaluronic acid (HA) is recognized as an anti-inflammatory, anti-edematous, antioxidant, bacteriostatic biomolecule with osteoinductive properties [14]. Thus, HA was proposed as adjunct to surgical and non-surgical periodontal therapies with the aim of enhancing clinical outcomes [15–17]. Findings have further confirmed the benefit of HA on diabetic wound healing [18]. To the best of our knowledge, HA local effect as an adjunct to PMPR was not yet assessed in diabetic type 2 stage-II periodontitis patients. Thus, the present randomized controlled trial aimed to evaluate the effect of topical HA gel with PMPR in diabetic type 2 stage-II periodontitis patients on clinical attachment level (CAL) (primary outcome), probing pocket depth (PPD), gingival recession depth (GRD), bleeding on probing (BOP), plaque index (PI), level of HbA1c, radiographic defect depth (DD), and radiographic bone density (RBD; all secondary outcomes). The null hypothesis formulated was that there would be no differences in CAL between the PMPR + HA and the PMPR groups.

## Methods

### Study design

Using the CONSORT guidelines, this study was designed as a six-months, parallel, prospective, randomized controlled trial [19]. On September 16, 2022, the trial protocol was registered on www.clinicaltrials.gov (ID: NCT05543434). On June 30, 2022, the Cairo University Faculty of Dentistry’s Ethics Committee for Scientific Research authorized the research protocol and the informed consent form on June 2022 (IRB:10|6|22). This study was conducted and reported in accordance with the Fortaleza 2013 revisions to the Helsinki Declaration of ethical standards and the EQUATOR criteria for medical research involving human subjects.

### Study population

From January till October 2023, the trial participants were recruited from the outpatient clinic of the department of Oral Medicine and Periodontology at Cairo University’s Faculty of Dentistry, Egypt. The screening procedure was done until the desired sample size was attained. This clinical trial involved patients who consented to participate and were clearly informed about the specific procedures and time frame. Participants in the trial provided written consent. Inclusion criteria for patients who were between the ages of 18 and 65 years, with no previous history of periodontal treatment, controlled type 2 DM (HbA1c < 7%) [20], stage-II grade B periodontitis [21], PPD greater than 3 mm at least at two nonadjacent teeth [22], non-smokers [22], and patients cooperative and willing to attend follow-up appointments. Pregnant or lactating females [23], individuals receiving orthodontic treatment, patients under anti-inflammatory or antimicrobial medications [24], patients with a history of using mouthrinses and patients who had any documented allergies were not included.

### Sample size

Based on a previous study [16], a mean CAL and standard deviation (SD) of 3.27 ± 1.64 mm and 1.63 ± 0.55 mm, for the test and control groups respectively, were used for sample size calculation. 1.34 was the effect size utilized in the computation, with α = 5% and β = 0.8. The calculation resulted in ten patients per group, which were increased to 13 patients (*n* = 13/group) to account for a possible 25–30% drop-out. G*Power software (University of Düsseldorf, Germany) was used for the sample size computation.

### Randomization and allocation concealment

Subjects who met the inclusion criteria and provided an informed approval were randomly distributed in a ratio of 1:1 to receive either PMPR (control-group) or PMPR with HA (test-group), using www.randomizer.org. The sequence was concealed in opaque and sealed envelopes. Following PMPR, the allocation was revealed (KFE) and participants were allocated to either test or control groups.

### Procedure

All patients received Step 1 periodontal therapy prior to the initiation of the experimental protocol, to ensure uniform baseline conditions for evaluating the efficacy of the intervention. The therapy included oral hygiene instruction and PMPR performed using ultrasonic and/or hand instruments. Additionally, patients received counseling on their risk factors and correction of iatrogenic factors that could hinder plaque removal. A re-evaluation was conducted after 6 weeks, and only patients who demonstrated adequate plaque control and met all inclusion criteria proceeded to the experimental phase.

**Fig. 1:**
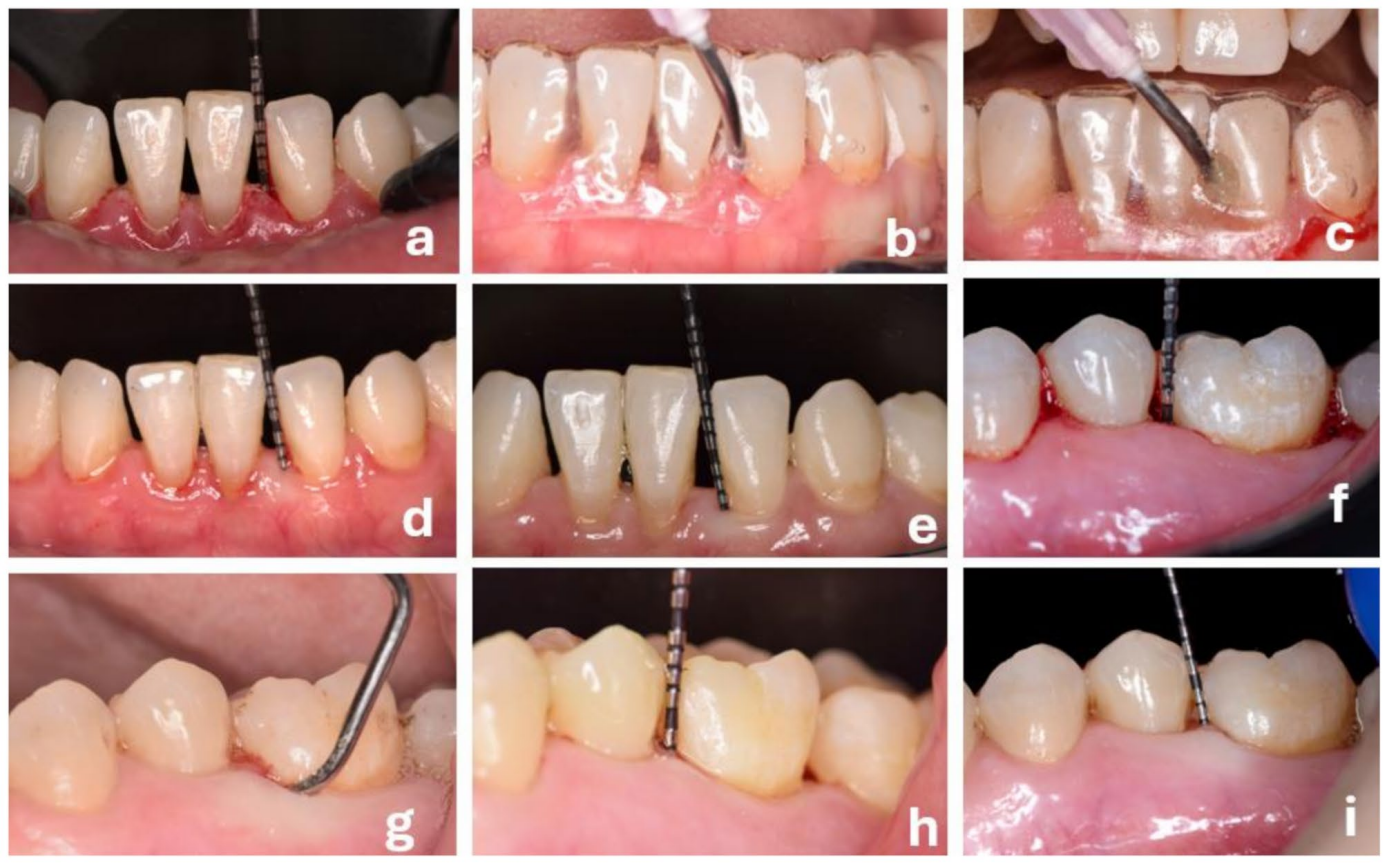
Clinical steps in representative case (**a-g**). (**a**) test case with 4 mm PPD with 3 mm with CAL at baseline. (**b**) application of topical hyaluronic acid gel using a stent in the pocket after therapy (**c**) Placement the stent again for 30 min one week following therapy (**d**) 2 mm PPD with 2 mm CAL at 2 months (**e**) 2 mm PPD with 2 mm CAL at 6 months. (**f**) control case with 5 mm PPD with 4 mm with CAL at baseline (**g**) SRP using Gracey curette (**h**) 3 mm PPD with 3 mm CAL at 3 months (**i**) 2 mm PPD with 2 mm CAL at 6 months

After administrating local anaesthetic (2% mepivacaine HCl with 1:100,000 adrenaline, Septodont, Saint-maur-des-fossés, France), patients in both groups underwent full mouth supra- and subgingival PMPR using ultrasonic scalers (Woodpecker UDS-P with LED, Guangxi, China) and hand Gracey curettes (Hu-Friedy, Chicago, IL, USA). Clinical and radiographic outcomes were measured and HbA1c levels were recorded (baseline). In the PMPR + HA group individualized acrylic stents were constructed on study models obtained from each patient a priori, with a circular opening placed at the site of all teeth with PPD > 3 mm [22] in each patient to apply HA gel (EZ-Cure Hyaluronic acid 0.88%, VITA, Mumbai, India) into the pocket immediately and after a week. Before applying the gel subgingivally via the syringe and a disposable blunt needle (Fig. 1), using cotton rolls, teeth were isolated and thoroughly dried via gentle air-drying [25]. The stent was left in place for 30 min, before the extra gel was removed with a cotton roll. All treatments were conducted by a single clinician (RA).

### Postoperative care

Patients were advised not to chewy and sticky items [26]. The patient did not receive any antiplaque agents, systemic antibiotics, or anti-inflammatory medications [27]. In addition, patients received instructions not to utilize aggressive dental cleaning agents, such as vigorous brushing or flossing, in the areas where the hyaluronic acid gel was applied in the two days following the treatment, that might interfere with the application of the gel. Follow-up visits were scheduled for both three and six months after the procedure. At these follow-up appointments, supragingival PMPR was conducted using ultrasonic scalers (Woodpecker), the levels of HbA1c were recorded and clinical and radiographic outcomes were measured.

### Outcomes

An UNC 15 periodontal probe (Nordent, IL, USA) was used to measure all clinical variable at baseline, three and six-months. All teeth with PPD > 3 mm in each patient were evaluated at six sites per patient (mesio-buccal, buccal, disto-buccal, mesio-lingual, lingual, and disto-lingual) and averaged. The patient served as the unit of evaluation. CAL was determined as the distance between the gingival sulcus bottom and the cemento-enamel-junction (CEJ) in mm [28], PPD was measured in mm from the margin of the gingiva to the periodontal pocket’s base [29], GRD was recorded as a measurement made from the greatest apical extension of the CEJ to the gingival margin [29], BOP was measured as bleeding positivity within 10 s after probing, expressed as a percentage (%) [30], PI was presented with a chart that indicates the presence of plaque throughout the mouth, along with the precise percentage (%) of plaque detected in the oral cavity [31].

Standardized radiographs were taken in each patient for the teeth exhibiting the deepest probing depths, which subsequently served as either test or control teeth within the trial, using the Heliodent Plus system at 60 kVp, 8 mA, and 0.10 s, utilizing the XCP X-ray holder kit (Dentsply Sirona, Charlotte, USA) and PSP sensor size two (Xios AE, Dentsply Sirona). For each patient, an individualized rubber bite block was constructed around the film holder for standardized radiographic registration using the paralleling technique at baseline, three, and six months.

**Fig. 2:**
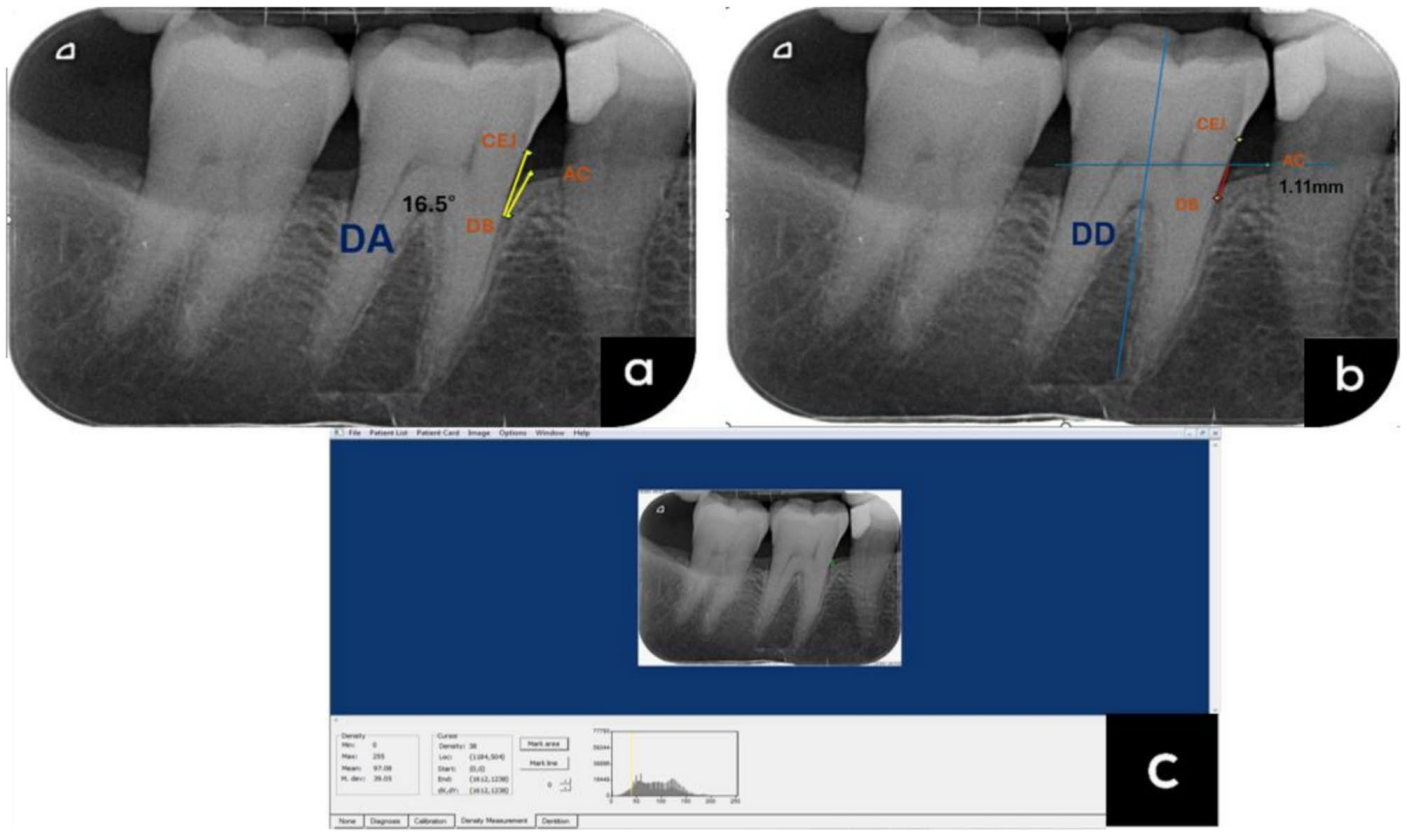
Digitized radiographic images (**a**) showing bone angle baseline measurements using ImageJ software (**b**) bone defects and the recorded linear measurements using ImageJ software (**c**) bone defect density measurement using Digora software

Using Image J software (Research Services Branch, NIH, Bethesda, Maryland, USA), three points were marked on each radiographic defect for the pockets exhibiting the deepest probing depths in each patient: the cementoenamel junction (CEJ), the alveolar crest (AC), and the bone defect’s deepest section (DB). The defect depth (DD) was measured at baseline, three, and six-months, from the AC to the DB. DD was calculated as the length of the DB-CEJ line measured at baseline and follow-up radiographs from DB to the point where the line between AC and the long axis of the tooth line intersects [32]. Defect angle was measured as the angle formed between the lateral defect boundary and the lines joining CEJ to DB at baseline [33]. Radiographic bone density (RBD) was measured in the interested area (ROI) outlined to include the defect’s limits with no superimposition of any portion of the tooth, and the mean grey values were then calculated. The ROI traced at baseline was superimposed on the three- and six-months radiographs, and changes in grey values were computed [34] (Fig. 2).

### Blinding

The outcome assessors (NS and AN) as well as the biostatistician were blinded. It was not feasible for the participants nor the principal investigator (RA) to be blinded, since participants in the PMPR + HA group received a different intervention.

### Calibration

All parameters were gathered by two experienced investigators (NS and AN) who were blinded. Before the study began, calibration was performed by comparing two measurements made by the two investigators on the same individuals (who were not part of the study one week apart) in order to ensure that the inter-examiner agreement for clinical and radiographic measurements was greater than 0.85.

### Statistical analysis

Numerical data were explored for normality by checking the distribution of data and using tests of normality (Kolmogorov-Smirnov and Shapiro-Wilk tests). All data showed non-normal (non-parametric) distribution while CAL, PPD, radiographic bone density (RBD) and blood glucose level (HBA1c) data showed normal (parametric) distribution. Data were presented as median, range, mean and standard deviation (SD) values. For non-parametric data, Mann-Whitney U test was used to compare between the two groups. Friedman’s test was used to study the changes by time within each group. Dunn’s test was used for pair-wise comparisons when Friedman’s test is significant. For parametric data, repeated measures ANOVA test was used to compare between the two groups as well as to study the changes by time within each group. Bonferroni’s post-hoc test was used for pair-wise comparisons when ANOVA test is significant. Qualitative data were presented as frequencies and percentages. Fisher’s Exact test was employed to compare between the two groups. Stepwise linear regression model was constructed to determine significant predictors of CAL after six months using gender, treatment (group), PPD, PI, GRD, defect angle, defect depth at baseline, HBA1c as independent variables at baseline and six months later. A significant threshold of *P* < 0.05 was established. IBM SPSS Statistics for Windows, Version 23.0, was used in order to do the statistical analysis. NY: IBM Corp., Armonk.

## Results

### Patient characteristics

**Fig. 3:**
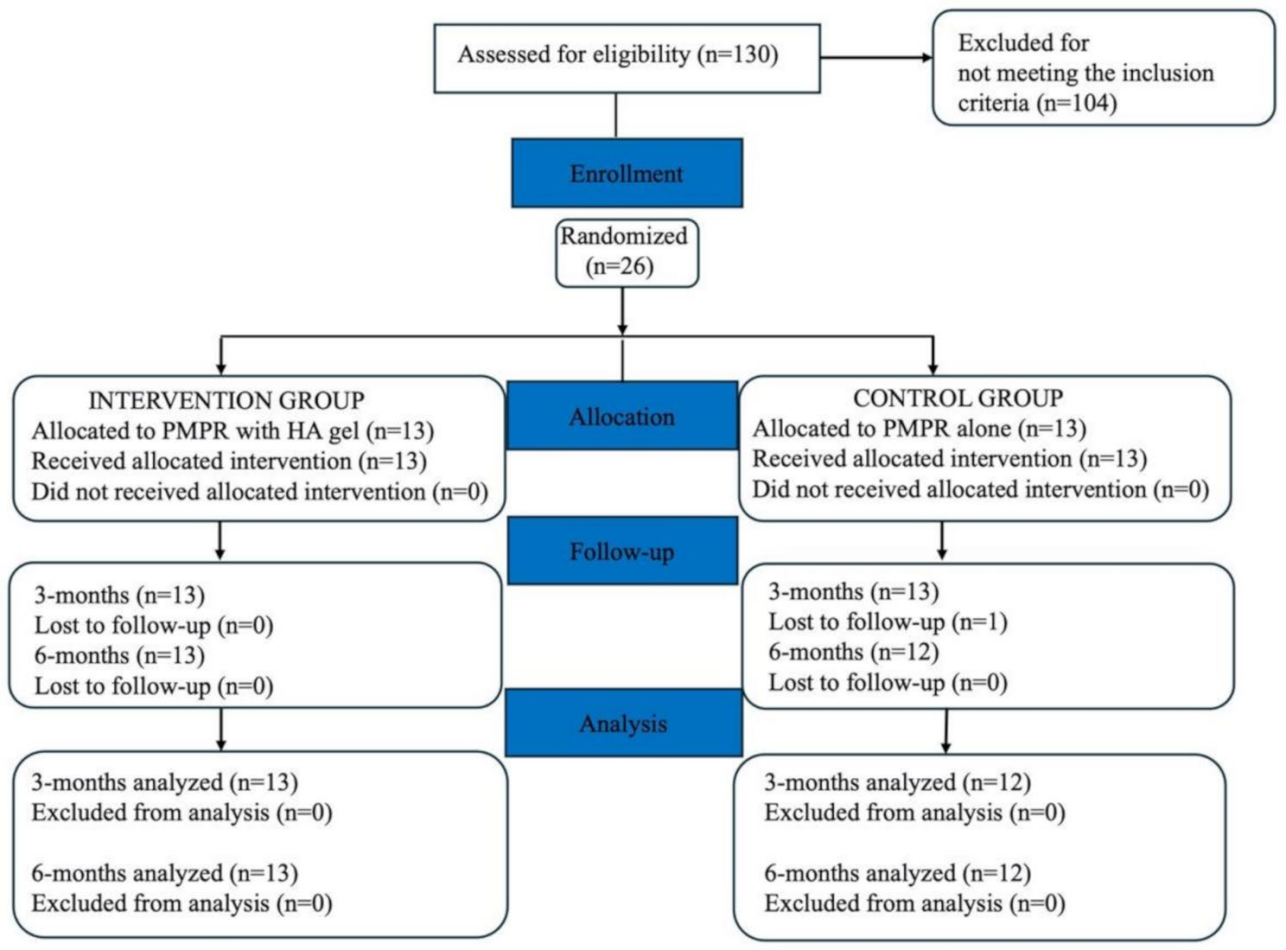
CONSORT flow diagram of participant recruitment

26 participants, were randomly categorized into the PMPR (*n* = 13; control-group) or the PMPR + HA (*n* = 13; test-group) (patient recruitment Fig. 3), with no significant differences in age, gender and DA at baseline (*p* > 0.05; Table 1). No inadvertent events occurred in any of the treated sites. All patients completed the follow up period, except for one patient in the control group (PMPR, female), who dropped out at three months.

**Table 1:** Baseline characteristics of age, gender, and radiographic defect angle in two groups (*n* = 13/group)

Variables	PMPR	PMPR + HA	*p*-value
Gender			
Females	12 females	11 females	1
Male	1 males	2 males	
Mean age ± SD (years)	45.2 ± 7.3	51.8 ± 9.2	0.051
Radiographic defect angle	23 ± 10.7	23.3 ± 11.8	0.980

PMPR, professional mechanical plaque removal, HA, hyaluronic acid, SD, standard deviation

### Clinical outcomes

No significant intergroup differences were notable over the study period (*p* > 0.05). Yet, both groups independently demonstrated significant improvement in the clinical periodontal parameters CAL, PPD, BOP and PI scores at six months in comparison to baseline (*p* < 0.05). CAL (mean ± SD) in the PMPR group decreased from 3.75 ± 0.45 mm to 2.75 ± 0.62 mm and in the PMPR + HA group from 3.77 ± 0.44 mm to 2.62 ± 0.51 mm, respectively. PPD was reduced from 4.75 ± 0.45 mm to 3.75 ± 0.45 mm in the PMPR group and from 4.85 ± 0.38 mm to 3.54 ± 0.52 mm in the PMPR + HA group. BOP was reduced from 66.2 ± 14.2% to 15.2 ± 6.5% in the PMPR group and from 66.6 ± 14.4% to 14.4 ± 6% in PMPR + HA group, respectively. PI reduced from 74.7 ± 16% to 20.3 ± 6.3% in the PMPR group and from 77.8 ± 9.5% to 19.1 ± 5.2% in the PMPR + HA group, respectively. Although in the PMPR + HA group a significant reduction HbA1c was evident at three and six months (*p* < 0.05), no significant improvement in HbA1c values was notable in the PMPR group (*p* > 0.05). Neither group showed significant improvements for GRD (*p* > 0.05; Table 2).

**Table 2:** Post-intervention changes in clinical variables at different follow-up visits

Variables	PMPR (*n* = 12)	PMPR + HA (*n* = 13)	*p*-value (Between groups)
Mean ± SD CAL (0 m) (mm)	3.75 ± 0.45 ^A^	3.77 ± 0.44 ^A^	0.92
Mean ± SD CAL (3 m) (mm)	2.92 ± 0.51 ^B^	2.77 ± 0.6 ^B^	0.52
Mean ± SD CAL (6 m) (mm)	2.75 ± 0.62 ^B^	2.62 ± 0.51 ^B^	0.56
*p*-value (Within group)	< 0.001*	< 0.001*	
Mean ± SD CAL change (3 m)(mm)	0.83 ± 0.39	1 ± 0.41	0.31
Mean ± SD CAL change (6 m) (mm)	1 ± 0.43	1.15 ± 1.20	0.35
Mean ± SD PPD (0 m) (mm)	4.75 ± 0.45 ^A^	4.85 ± 0.38 ^A^	0.57
Mean ± SD PPD (3 m) (mm)	3.92 ± 0.51 ^B^	3.69 ± 0.63 ^B^	0.34
Mean ± SD PPD (6 m) (mm)	3.75 ± 0.45 ^B^	3.54 ± 0.52 ^B^	0.29
*p*-value (Within group)	< 0.001*	< 0.001*	
Mean ± SD PPD reduction (3 m) (mm)	0.83 ± 0.39	1.15 ± 0.55	0.11
Mean ± SD PPD reduction (6 m) (mm)	1 ± 0.43	1 ± 1.20	0.11
Mean ± SD GRD (0 m) (mm)	1 ± 0.58	1.08 ± 0.49	0.72
Mean ± SD GRD (3 m) (mm)	1 ± 0.58	0.92 ± 0.28	0.68
Mean ± SD GRD (6 m) (mm)	1 ± 0.58	1 ± 0.60	0.69
*p*-value (Within group)	constant	0.135	
Mean ± SD BOP% (0 m)	66.2 ± 14.2 ^A^	66.6 ± 14.4 ^A^	0.7
Mean ± SD BOP% (3 m)	21.9 ± 6.4 ^B^	17.6 ± 8.3 ^B^	0.21
Mean ± SD BOP% (6 m)	15.2 ± 6.5 ^C^	14.4 ± 6.0 ^B^	0.81
*p*-value (Within group)	< 0.001*	< 0.001*	
Mean ± SD PI % (0 m)	74.7 ± 16.0 ^A^	77.8 ± 9.50 ^A^	0.68
Mean ± SD PI % (3 m)	29.9 ± 6.80 ^B^	22.3 ± 9.40 ^B^	0.04*
Mean ± SD PI % (6 m)	20.3 ± 6.30 ^C^	19.1 ± 5.20 ^B^	0.74
*p*-value (Within group)	< 0.001*	< 0.001*	
Mean ± SD HbA1c% (0 m)	6.58 ± 0.36	6.58 ± 0.36 ^A^	0.95
Mean ± SD HbA1c% (3 m)	6.41± 0.37	6.40 ± 0.34 ^B^	0.95
Mean ± SD HbA1c% (6 m)	6.43 ± 0.33	6.45 ± 0.37 ^B^	0.99
*p*-value (Within group)	0.067	0.032*	

PMPR, professional mechanical plaque removal, HA, hyaluronic acid, SD, standard deviation, GRD: Gingival recession depth, 0 m = baseline, 1 m = 1-month, 3 m = 3-months, 6 m = 6-months

* Significant at *p* ≤ 0.05. Different superscripts in each column represent changes within each group

### Radiographic outcomes

No significant intergroup differences were notable for DD and RBD (*p* > 0.05). Yet, both groups independently demonstrated significant improvement in DD and RBD (*p* < 0.05). Although the PMPR group demonstrates no significant change in DD and RBD at three-months (*p* > 0.05), a significant improvement was observed between three and six-months (*p* < 0.05). In contrast, the PMPR + HA group showed a significant reduction in DD with an increase in RBD after three as well as from three to six months (*p* < 0.05; Table 3).

**Table 3:** Comparison between radiographic variables at different follow-up visits as well as the changes within each group

Variables	PMPR (*n* = 12)	PMPR + HA (*n* = 13)	*p*-value (Between groups)
Mean ± SD DD (0 m) (mm)	1.45 ± 0.44 ^A^	1.47 ± 1.10 ^A^	0.27
Mean ± SD DD (3 m) (mm)	1.37 ± 0.40 ^A^	1.20 ± 1.67 ^B^	0.14
Mean ± SD DD (6 m) (mm)	1.25 ± 0.38 ^B^	0.97 ± 0.38 ^C^	0.07
*p*-value	0.005*	0.002*	
Mean ± SD BD (0 m) (mm)	46.80 ± 10.30 ^B^	46.80 ± 13.90 ^C^	0.99
Mean ± SD BD (3 m) (mm)	50.80 ± 8.80 ^B^	54.10 ± 15.20 ^B^	0.52
Mean ± SD BD (6 m) (mm)	59.60 ± 7.00 ^A^	61.20 ± 16.20 ^A^	0.76
*p*-value	0.001*	0.001*	

PMPR, professional mechanical plaque removal; DD, defect depth; BD, bone density, SD, standard deviation 3 m = 3-months, 6 m = 6-months

* Significant at *p* ≤ 0.05

### Stepwise linear regression analysis

Regression analysis results revealed that baseline GRD, PPD, DD were the sole significant predictor of CAL six months later, with an inverse correlation between baseline GRD, DD and a direct correlation of baseline PPD with CAL after six months (*p* < 0.05; Table 4).

**Table 4:** Results of Stepwise linear regression analysis model showing predictors of CAL after six months

Variables	*B*	SE	*p*-value	95% CI for *B*	
GRD at base line	-1.01	0.17	< 0.001*	-1.36	-0.67
PPD at base line	0.794	0.22	0.001*	0.345	1.243
DD at base line	-0.24	0.09	0.011*	-0.42	-0.06

*: Significant at *p* ≤ 0.05, SE: Standard Error, CI, confidence interval; *B*, regression coefficient

## Discussion

Professional mechanical plaque removal (PMPR) represents an effective treatment modality for pocket reduction (in depth and number) in periodontal disease [35–40]. Yet, under certain challenging clinical conditions, including patients with DM, the response of the periodontal pockets to PMPR might not be sufficient clinically [41]. Thus, various local adjunctive agents, including HA [42–44] and its modifications [45], were advocated to foster the PMPR’s outcome [8, 25, 46–49]. Local satranidazole gel subgingival application as an adjunct to PMPR reduced PPD in type 2 DM periodontitis patients compared to PMPR alone. Doxycycline gel, chlorhexidine gel, and tetracycline fiber improved HbA1c values in in type 2 DM periodontitis patients over three months [50]. Local administration of metformin or statins to PMPR improved PPD and CAL compared to PMPR alone [51]. Adjunctive i-PRF to PMPR improved periodontal parameters in type 2 DM periodontitis patients compared to PMPR with saline as a placebo [51]. Furthermore, the adjunctive application of ozone gel to PMPR resulted in significant reduction in PPD, plaque and bleeding indices and CAL-gain, compared to PMPR alone in type 2 DM periodontitis patients [25]. The addition of melatonin, resveratrol, omega-3 fatty acids and cranberry juice, propolis and aloe vera gel to periodontal therapy demonstrated a significant improvement in periodontal parameters in type 2 DM periodontitis patients [52].

Similar to a recent three-months trial [53], PMPR was found to improve HbA1c levels in diabetic patients at three months [54, 55] with baseline HbA1c levels affecting its therapeutic reduction [56]. Findings further validated that HA could exert beneficial effects on periodontal therapy [16, 57–62] and inflammation [63, 64]. HA is believed to influence early wound healing events, particularly cellular proliferation and the maturation of granulation tissue. A majority of its positive attributes is believed to rely on its ability in providing a scaffold for cellular attachment and migration as well as extracellular matrix [65] and bone formation [66]. Thus, the current randomized trial examined for the first time the possible adjunctive effects of HA on PMPR in individuals with controlled type 2 diabetes and stage-II grade B periodontitis.

In accordance with earlier findings on the positive effect of PMPR in diabetic periodontitis patients [67–69], in the current trial, CAL, PPD, BOP, PI, DD and RBD significantly improved in both groups independently, with no significant intergroup differences over six months. PPD, GRD, PPD and DD at baseline were significant predictors for CAL after six months. There was inverse correlation between baseline GRD and DD as well as a direct correlation of baseline PPD with CAL after six months, suggesting that patients with lower radiographic defect depth and gingival recession and higher pocket depth at the start of the treatment tended to have greater improvement in CAL. Meanwhile, the absence of an additional favorable effect of the local HA application is in accordance with an earlier investigation employing HA as an adjunct to PMPR in patients with severe periodontitis [70] and smoking [71]. Similar to smokers, patients with diabetes mellitus (DM) might have a systemically impaired ability, due to the cumulative biological damage in their tissues, to favorably respond to a locally adjunctive HA application, for significantly improving their periodontal outcomes [72]. Yet, it should be noted that stage-II periodontitis patients of the current trial exhibited shallow periodontal pockets. In contrast, local HA application was shown to improve PPD and CAL by an average of 1 mm in type 2 diabetic patients with deeper periodontal pockets (type III or IV periodontitis) [53].

HbA1c was one of the present trial’s secondary outcomes and accordingly the study was not powered for this metabolic endpoint determination. Yet, interestingly the current trial demonstrated, for the first time, that the local application of HA as an adjunct to PMPR in the controlled type 2 DM patients had a positive effect on HbA1c. Although clinically of low significance, a statistically significant improvement of 0.13% was notable in the HbA1c values of the PMPR + HA group over six months. A previous investigation testing HA-injections in non-surgical treatment of knee injuries demonstrated that patient expectations and psychological stress are believed to affect treatment outcomes, with greater observed effects [73]. Thus, it is plausible to similarly assume that the currently observed positive impact on HbA1c could be attributed to the Hawthorne effect, inducing an increased patient education, commitment, patients’ compliance with their metabolic control regimen and motivation (all pivotal in the initial S0 and S1 steps of periodontal therapy), in the group receiving the HA gel with a potential link to enhanced oral hygiene, lifestyle behavior, and diet control [74, 75]. It can be further hypothesized, that the reduction in the HbA1c values of the patients in the PMPR + HA group, similar to previous findings [76–79], might be attributed to their motivation and possibly improved adherence to a healthier lifestyle, an attitude, which, in comparison to the PMPR group, may have been fostered by the increased attention they received, being selected to receive the HA application, and their appreciation of the possibility of an adjunctive effect of the local HA application and their lifestyle change on their oral and diabetic status. Further, this finding on HbA1c could suggest a potential short-term benefit of local HA adjunctive to PMPR in improving glycemic control, being anti-inflammatory and anti-edematous, potentially improving the patients’ insulin sensitivity. All these factors could have acted in combination or isolated to result in the observed improvement.

Nevertheless, it is necessary to recognize the study’s limitations, including the absence of the measurement patient related outcomes. Second, although a sample size calculation was conducted, a larger sample size could have improved the external validity (generalizability) of the study findings. Third, it was not easy to enroll controlled type 2 DM patients, meeting the inclusion criteria, while additionally having low PI. Fourth, higher concentrations or varied formulations of locally applied HA could have yielded different results. Fifth, although a six-months follow up is generally acceptable to observe changes in outcomes following non-surgical periodontal therapy, a longer follow up could have given better insights to assess the long-term effects on the metabolic and periodontal levels. Sixth, the included patients stemmed from populations experiencing socioeconomic challenges, which could have further affected the life style, oral health habits and the trial’s outcomes. Finally, future studies could incorporate specific measures to quantify patient engagement and adherence in the context of the Hawthorne effect, which might shed more light on the contribution of such behavioral factors on metabolic changes and periodontal outcomes.

## Conclusions

Based on current findings, stage-II periodontitis patients with controlled type 2 DM responded favorably to PMPR, with or without local HA gel application. Interestingly, HA as an adjunct to PMPR significantly improved HbA1c levels in controlled type 2 diabetic stage-II periodontitis patients. Additional longitudinal clinical trials with larger sample sizes, longer follow ups and patients of various socioeconomic standards should be conducted to fully investigate the potential impact of local HA application in controlled diabetic periodontitis patients.

## Data Availability

The datasets used and/or analysed during the current study are available from the corresponding author on reasonable request.

## Abbreviations

BD: Bone density
BOP: Bleeding on probing
CAL: Clinical attachment level
DD: Defect depth
GRD: Gingival recession depth
HbA1c%: Glycosylated hemoglobin percentage
NSPT: Non-surgical periodontal therapy
PI: Plaque index
PMPR: Professional mechanical plaque removal
PMPR: Professional mechanical plaque removal
PPD: Probing pocket depth
